# Phylogenomic analysis demonstrates a pattern of rare and long-lasting concerted evolution in prokaryotes

**DOI:** 10.1038/s42003-018-0014-x

**Published:** 2018-02-08

**Authors:** Sishuo Wang, Youhua Chen

**Affiliations:** 1 Beaty Biodiversity Research Centre, University of British Columbia, 2212 Main Mall, Vancouver, BC V6T 1Z4 Canada; 2 Department of Botany, Faculty of Science, University of British Columbia, 3529-6270 University Boulevard, Vancouver, BC V6T 1Z4 Canada; 3 Chengdu Institute of Biology, Chinese Academy of Sciences, 610000 Chengdu, China; 4 Department of Renewable Resources, Faculty of Agricultural, Life and Environmental Sciences, University of Alberta, Edmonton, AB T6G 2H1 Canada

## Abstract

Concerted evolution, where paralogs in the same species show higher sequence similarity to each other than to orthologs in other species, is widely found in many species. However, cases of concerted evolution that last for hundreds of millions of years are very rare. By genome-wide analysis of a broad selection of prokaryotes, we provide strong evidence of recurrent concerted evolution in 26 genes, most of which have lasted more than ~500 million years. We find that most concertedly evolving genes are key members of important pathways, and encode proteins from the same complexes and/or pathways, suggesting coevolution of genes via concerted evolution to maintain gene balance. We also present LRCE-DB, a comprehensive online repository of long-lasting concerted evolution. Collectively, our study reveals that although most duplicated genes may diverge in sequence over a long period, on rare occasions this constraint can be breached, leading to unexpected long-lasting concerted evolution in a recurrent manner.

## Abstract (web summary)

Sishuo Wang and Youhua Chen present an analysis of concerted evolution in prokaryotes using a new computational pipeline, iSeeCE. They find evidence in 26 genes for recurrent concerted evolution, most of which last more than ~500 million years, and provide a database, LRCE-DB, for data exploration.

## Introduction

Gene duplication is a key force in driving gene evolution as evident from the prevalence of duplicated genes in almost all sequenced species^1,2^. Traditionally, theories of population genetics predict that entirely redundant duplicates cannot be retained in the genome over time^3^. Indeed, duplicated genes that are stably preserved in the genome for a long time often diverge in sequence, expression or function^2,4,5^. In some cases, duplicated genes may display concerted evolution where paralogs within the same species show more similar sequences than orthologs in other species, which usually results from gene conversion or unequal recombination^6,7^.

Concerted evolution has been found in the evolution of many genes in both prokaryotes and eukaryotes, and is most often observed in rRNAs^6,7^. However, repeated concerted evolution of protein-coding genes across species is mostly found to occur on relatively short time scales; the evidence for those that last for hundreds of millions of years is very rare^7–9^. For example, the duration of the concerted evolution of genes derived from the whole-genome duplication event in budding yeast was estimated to be around 25 Ma (million years)^10^, with the exception of ribosomal protein genes, which have likely undergone concerted evolution since the whole-genome duplication (~100 Ma)^11^. Wang et al.^12^ summarized the duration of multiple previously reported concerted evolution events, and found that most of them last for no more than 100 Ma. One well-documented example of long-lasting concerted evolution is *tuf*, the gene coding for the elongation factor tu, which was found to experience frequent concerted evolution in a large number of species in Proteobacteria^13,14^. *mtrA*, a gene crucial to methanogenesis, was also observed to have undergone concerted evolution since the divergence of many methanogens^12^.

Concertedly evolving paralogs from the same species show higher sequence similarity to each other than either does to orthologs in other species, and often form monophyly in the phylogenetic tree. However, such a pattern could also arise from lineage-specific gene duplication. To distinguish between these two scenarios, it is very important to take gene synteny into consideration to resolve the orthology and paralogy of the gene^7,15^. This is because paralogs with shared synteny across species are unlikely to be derived from independent gene duplication, and thereby should result from concerted evolution^15,16^.

To investigate the long-term impact and facilitate the genome-wide identification of concerted evolution, we developed a comprehensive bioinformatic pipeline, iSeeCE, which integrates the information of both phylogeny and synteny in the analysis. We applied it to identify long-lasting recurrent concerted evolution in a broad range of prokaryotes. We analyzed the functions of concertedly evolving genes, and discussed the potential driving forces underlying the recurrent concerted evolution over such a long period. Finally, we developed an online database LRCE-DB (www.lrgcdb.eu) to provide a user-friendly interface for researchers to explore the data.

## Results

### Identification of long-lasting recurrent concerted evolution

**Fig. 1:**
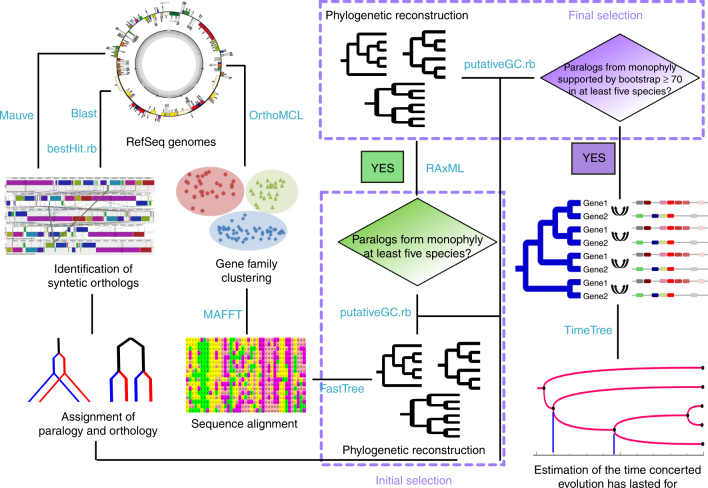
The schematic diagram detailing the phylogenomic approach of the identification of recurrent concerted evolution in prokaryotes

Much of the difficulty in inferring concerted evolution results from the lack of gene synteny information and accuracy of phylogeny. iSeeCE (Fig. 1; full implementation available at https://github.com/evolbeginner/iSeeCE), presented in this study, addressed the above challenges by integrating the information of gene synteny across species to accurately assign the orthology and paralogy relationships of genes, performing two rounds of phylogenetic reconstructions, and automatically parsing the results in a high-throughput way (see Methods; Fig. 1; Supplementary Fig. 1). We identified concertedly evolving genes in the unit of order. We applied iSeeCE to the identification of concerted evolution in 69 orders of prokaryotes including 682 carefully selected species (see Methods). Only genes that displayed patterns of concerted evolution in at least five different species were considered as genes undergoing recurrent concerted evolution (see Methods).

In total, we detected 19 and 7 genes that undergo recurrent concerted evolution in bacteria and archaea, respectively (Tables 1 and 2). *tuf* and *mtrA*, the two genes that were previously reported to have undergone long-lasting recurrent concerted evolution^12,14^, were successfully detected using our computational framework. The vast majority of concerted evolution events identified here occurred in species from a single order (Tables 1 and 2). Two genes were found to evolve concertedly in two orders (Tables 1 and 2). One gene (*tuf*) was found to experience concerted evolution in species from 29 orders.

**Table 1:** Genes undergoing long-lasting recurrent conversion in bacteria

Gene	Function	Phylum	Order	No. of species	Duration (Ma)	Complex
*nuoL*	NADH-quinone oxidoreductase subunit L	Aquificae	Aquificales	6	2075	Complex I
*Dxs*	1-deoxy-D-xylulose-5-phosphate synthase	Alpha-proteobacteria	Rhodospirillales	5	48	DXS
*psbA*	Photosystem II q(b) protein	Cyanobacteria	Nostocales, Oscillatoriales	13	2322	PS II
*psbD*	Photosystem II q(a) protein	Cyanobacteria	Nostocales, Synechococcales, Oscillatoriales, Chroococcales	20	2594	PS II
*ftsH*	ATP-dependent zinc metalloprotease FtsH	Deinococcus-Thermus	Deinococcales	5	439	FtsH
*eftA*	Electron transfer flavoprotein subunit alpha	Beta-proteobacteria	Burkholderiales	10	936	ETF
*eftB*	Electron transfer flavoprotein subunit beta	Beta-proteobacteria	Burkholderiales	9	936	ETF
*amoA*	Ammonia monooxygenase subunit A	Beta-proteobacteria	Nitrosomonadales	8	449	AMO
*amoB*	Ammonia monooxygenase subunit B	Beta-proteobacteria	Nitrosomonadales	8	449	AMO
*amoC*	Ammonia monooxygenase subunit C	Beta-proteobacteria	Nitrosomonadales	8	449	AMO
*amoD*	Hypothetical protein	Beta-proteobacteria	Nitrosomonadales	8	449	N/A
*amoE*	Hypothetical protein	Beta-proteobacteria	Nitrosomonadales	8	449	N/A
*haoA*	Hydroxylamine reductase	Beta-proteobacteria	Nitrosomonadales	8	449	HAO/c554
*haoB*	Hydroxylamine oxidation protein HaoB	Beta-proteobacteria	Nitrosomonadales	8	449	N/A
*cycA*	Cytochrome c_554_	Beta-proteobacteria	Nitrosomonadales	8	449	HAO/c554
*cycB*	Cytochrome c_m552_	Beta-proteobacteria	Nitrosomonadales	8	449	Cyt c_m552_
*fla*	Flagellin	Gamma-proteobacteria	Alteromonadales	8	1733	Filament
*tkt*	Transketolase	Gamma-proteobacteria	Vibrionales	6	124	TKT
*tuf*	Elongation factor Tu	6 phyla	29 orders	221	3936	–

Protein functions are obtained from public databases (PDB, UniProt, BRENDA, MetaCyc, etc.) and literature

*Ma* million years

**Table 2:** Genes undergoing long-lasting recurrent conversion in archaea

Gene	Function	Phylum	Order	No. of species	Duration (Ma)	Complex
*mtmB*	Monomethylamine methyltransferase MtmB	Euryarchaeota	Methanosarcinales	7	496	MtmB-MtmC
*mtmC*	Monomethylamine corrinoid protein MtmC	Euryarchaeota	Methanosarcinales	6	496	MtmB-MtmC
*mtbB*	Dimethylamine methyltransferase MtbB	Euryarchaeota	Methanosarcinales	8	496	MtbB-MtbC
*mtbC*	Dimethylamine corrinoid protein MtbC	Euryarchaeota	Methanosarcinales	6	496	MtbB-MtbC
*mtrA*	Tetrahydromethanopterin S-methyltransferase subunit A	Euryarchaeota	Methanomicrobiales, Methanococcales	12	1943	Mtr
*glnB*	Nitrogen regulatory protein P-II	Euryarchaeota	Methanococcales	5	1943	GlnB
N/A	Archaeal histone	Euryarchaeota	Methanococcales	5	1183	Archaeal histone

Protein functions are obtained from public databases (PDB, UniProt, BRENDA, MetaCyc, etc.) and literature

*Ma* million years

Recurrent concerted evolution should start prior to the divergence of the species where concerted evolution is detected^13,17^. For each concertedly evolving gene, we estimated the minimum duration it has lasted for based on the divergence time of species provided by TimeTree. The mean and median of the lasting time of identified concerted evolution are 1018 Ma and 496 Ma, respectively (Tables 1 and 2). Eight genes have evolved in a concerted manner for more than 1000 Ma. The above results reveal the longer-lasting effects of concerted evolution on gene evolution than previously appreciated. Also, the high sequence identity between paralogs undergoing concerted evolution across nearly the full length of the gene indicates that the process is still ongoing in most identified genes (for alignments see www.lrgcdb.eu/Tree.php).

### Concerted evolution of genes in ammonia oxidation pathway

**Fig. 2:**
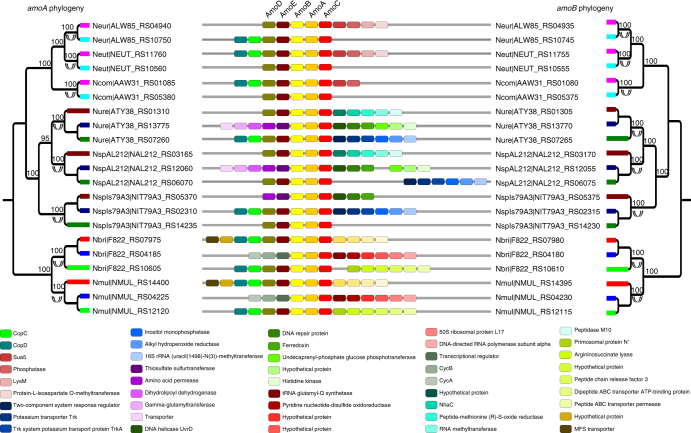
Phylogenetic trees of *amoA* and *amoB* in Nitrosomonadales. Double-headed arrows indicate concerted evolution events. Syntenic orthologs are represented by thick branches in the same color in the phylogeny. Flanking genes are denoted by colored bricks, and chromosome segments are denoted by gray bars. Genes involved in the ammonia oxidation pathway (*amoA-E*) are labeled above the colored bricks. The functions of flanking genes are shown at the bottom. Numbers adjacent to the nodes in the phylogeny are bootstrap percentages obtained from 500 pseudoreplicates. Only bootstrap percentages ≥50 are shown. The name of each operational taxonomic unit is represented by the abbreviation of species name and gene locus. Abbreviations of species names are listed in Supplementary Data 1

**Fig. 3:**
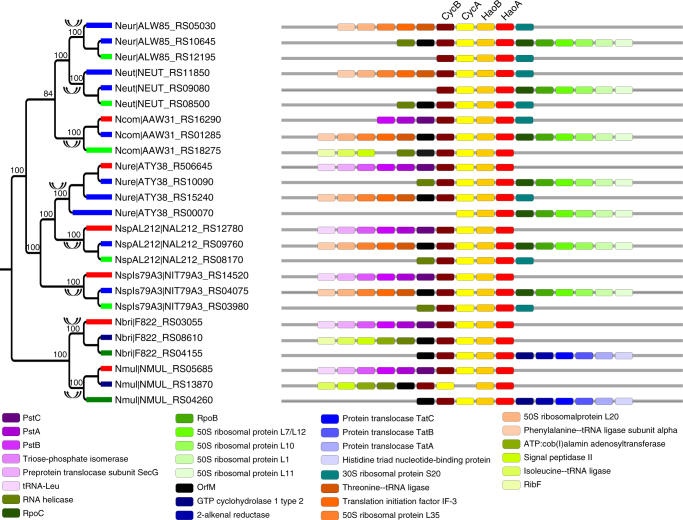
Phylogenetic trees of *haoA* in Nitrosomonadales. Double-headed arrows indicate concerted evolution events. Syntenic orthologs are represented by thick branches in the same color in the phylogeny. Flanking genes are denoted by colored bricks, and chromosome segments are denoted by gray bars. Genes involved in the ammonia oxidation pathway (*haoAB* and *cycAB*) are labeled above the colored bricks. The functions of flanking genes are shown at the bottom. Numbers adjacent to the nodes in the phylogeny are bootstrap percentages obtained from 500 pseudoreplicates. Only bootstrap percentages ≥50 are shown. The name of each operational taxonomic unit is represented by the abbreviation of species name and gene locus. Abbreviations of species names are listed in Supplementary Data 1

Intriguingly, all genes involved in ammonia oxidation, the first step of nitrification, were present in multiple copies with nearly identical nucleotide sequences in all of the eight analyzed species from Nitrosomonadales, a group of ammonia-oxidizing bacteria from Beta-proteobacteria. These genes are encoded by the operon *amoCAB* (ammonia monooxygenase), *haoAB* (hydroxylamine oxidoreductase), and *cycAB* (cytochrome c_554_ and c_m552_)^18^. Products of these genes constitute three protein complexes (AMO, HAO/c554 and cm552) that catalyze the conversion of ammonia (NH_3_) to nitrite (NO_2_-) (Supplementary Fig. 2), enabling ammonia-oxidizing bacteria to use energy from this reaction and causing nitrogen to enter the biosphere^19^. The other two genes, *amoD* and *amoE* (also known as *orf5* and *orf4*), are also considered to be involved in ammonia oxidation although their detailed functions are still unknown^20^. The presence of multiple copies of the operon *amoCAB* in the ammonia oxidation pathway in *Nitrosospira* sp. NpAV, a species from Nitrosomonadales, was first noticed by Norton et al. (1996), and was attributed to recent duplication due to the lack of genomic data available^21^. Through comprehensive analysis of the genomic context of eight Nitrosomonadales genomes, we found that the operons *amoCAB*, *haoAB*, and *cycAB* were surrounded by conserved gene synteny (Figs. 2, 3; Supplementary Fig. 3). This result ruled out the possibility that the observed topology of the phylogeny results from recent duplication in each species, as convergent duplication in the syntenic regions among different species is unlikely to happen^7,15^. Instead, the above results indicated that duplication of the nine genes occurred before the divergence of all or some of the species in the order Nitrosomonadales. Hence, these findings demonstrate recurrent concerted evolution of all genes participating in ammonia oxidation, which, to the best of our knowledge, represents the first case of concerted evolution of all genes of an entire pathway over such a long time. In addition, the extremely high sequence similarity between paralogs (Supplementary Fig. 4) indicates that the process of concerted evolution is still ongoing.

### Concertedly evolving genes are involved in important pathways

**Fig. 4:**
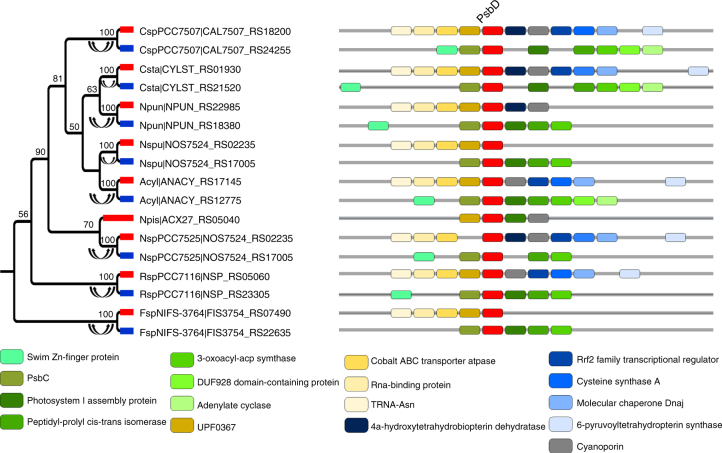
Phylogenetic trees of *psbD* (labeled at the top of the figure) in Nostocales from cyanobacteria. Double-headed arrows indicate concerted evolution events. Syntenic orthologs are represented by thick branches in the same color in the phylogeny. Flanking genes are denoted by colored bricks, and chromosome segments are denoted by gray bars. The functions of flanking genes are shown at the bottom. Numbers adjacent to the nodes in the phylogeny are bootstrap percentages obtained from 500 pseudoreplicates. Only bootstrap percentages ≥50 are shown. The name of each operational taxonomic unit is represented by the abbreviation of species name and gene locus. Abbreviations of species names are listed in Supplementary Data 1

Another interesting example of genes that undergo long-lasting concerted evolution is *psbA* and *psbD*, two homologous genes that comprise the reaction center of photosynthesis II (PS II) complex in cyanobacteria^22^. We found that most cyanobacteria species carried two copies of *psbD*. Genomic context analysis revealed two types of *psbD* with conserved synteny across species (Fig. 4; Supplementary Fig. 5). Phylogenetic analysis showed that paralogs from the same species often clustered in the same clade (Fig. 4; Supplementary Fig. 5). A similar pattern was also observed for *psbA* in Nostocales and Oscillatoriales (Supplementary Fig. 6a, b). These findings strongly indicated recurrent concerted evolution of *psbA* and *psbD* in cyanobacteria. Additionally, most species in the other two cyanobacterial lineages (Chroococcales and Synechococcales) possessed multiple copies of *psbA* with nearly identical sequences that clustered together in the gene tree without synteny detected (Supplementary Fig. 6c, d). It is possible that *psbA* paralogs evolved in a concerted manner in Chroococcales and Synechococcales but the synteny of their neighboring genes were disrupted due to genomic rearrangement.

The gene conversion of the two copies of *elongation factor tu* (*tufA* and *tufB*) was previously described in Proteobacteria, particularly Gamma-proteobacteria^13,14^. Here we examined the phylogeny of *tuf* with a much broader range of taxa. In addition to Proteobacteria, species from Aquificae, Acidobacteria, Actinobacteria, Chloroflexi, and Deinococcus–Thermus possessed two duplicates of *tuf* that had undergone recurrent concerted evolution. The two copies of *tuf* genes in different species were characterized by their different genomic contexts (Supplementary Fig. 7a). The phylogeny of *tuf* is basically consistent with the species phylogeny of bacteria (Supplementary Fig. 7b). These findings indicate that *tuf* was duplicated prior to the emergence of most extant bacterial lineages, followed by extensive gene conversions and multiple lineage-specific gene losses. Hence, the evolution of *tuf* likely represents the longest-lasting concerted evolution that has been identified so far (Table 1).

The other seven genes undergoing recurrent concerted evolution in bacteria also have important functions (Table 1). Among these genes, three, *nuoL, eftA*, and *eftB*, are involved in energy conversion, the latter two of which constitute the electron transfer flavoprotein (ETF), a heterodimer that transfers electrons to terminal respiratory systems^23^. Two genes, *tkt* and *dxs*, participate in carbohydrate metabolism^24,25^. *ftsH* plays a major role in the degradation and quality control of membrane proteins^26^. Encoded by *fla*, flagellin is the principal component of bacterial flagellum^27^.

**Fig. 5:**
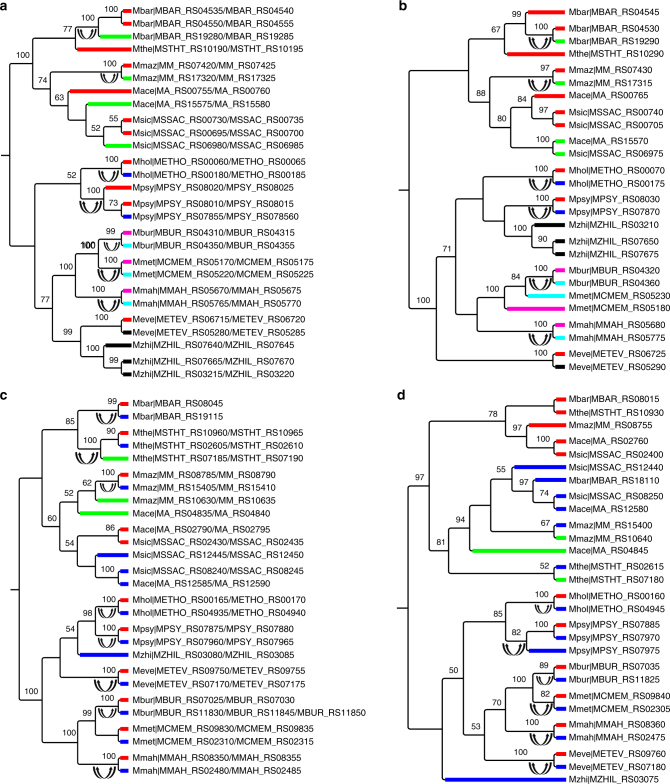
Phylogenetic trees of *mtmB* (**a**), *mtmC* (**b**), *mtbB* (**c**), and *mtbC* (**d**) in Methanosarcinales. Double-headed arrows indicate concerted evolution events. Syntenic orthologs are represented by thick branches in the same color in the phylogeny. Numbers adjacent to the nodes in the phylogeny are bootstrap percentages obtained from 500 pseudoreplicates. Only bootstrap percentages ≥50 are shown. The name of each operational taxonomic unit is represented by the abbreviation of species name and gene locus. Abbreviations of species names are listed in Supplementary Data 1

All of the seven concertedly evolving genes identified in archaea are from methanogenic species, among which five genes are involved in methanogenesis (Table 2). In addition to the previously reported *mtrA*^12^, a gene crucial to the hydrogenotrophic methanogenesis pathway, we identified recurrent concerted evolution in another four genes (*mtmB*, *mtmC*, *mtbB*, and *mtbC*) involved in the methylotrophic methanogenesis pathway (Fig. 5a–d). The methylotrophic pathways for methanogenesis from monomethylamine and dimethylamine are mainly found in Methanosarcinales^28^. They follow a similar route involving an enzyme system consisting of three proteins: a protein binding the corrinoid prosthetic group (encoded by *mtmC* or *mtbC*), and two methyltransferases, designated MT1 (encoded by *mtmB* or *mtbB*) and MT2 (encoded by *mtbA*)^29,30^. MT1 and the corrinoid protein form a tight complex and catalyze the transfer of the methyl group from the substrate to the corrinoid group, the first step of the whole pathway (Supplementary Fig. 8). These results suggest the important role of concerted evolution on the evolution of genes involved in the methane metabolism and energy conservation in archaea.

### Concerted evolution of genes in the same complexes/pathways

We found that 22 out of 26 genes that showed evidence of long-lasting recurrent concerted evolution identified in this study encode proteins in stable protein complexes (Tables 1 and 2). Intriguingly, among these 22 genes, 17 genes encode proteins that are from the same complexes and/or pathways. These genes include genes involved in the ammonia oxidation pathway (*amoCAB*, *amoDE*, *haoAB*, and *cycAB*), genes encoding the reaction center of photosystem II (PS II) (*psbA* and *psbD*), genes constituting the complex catalyzing methyl transfer from monomethylamine (*mtmBC*) and dimethylamine (*mtbBC*) in methanogenesis, and genes encoding the two subunits of bacterial electron transfer flavoprotein (*eftA* and *eftB*). These findings suggest the coadaptation and coevolution of genes encoding proteins in the same complexes and/or pathways via concerted evolution of paralogs.

In general, genes undergoing long-lasting concerted evolution play important roles in various biological pathways. This is likely different from genes undergoing short-term concerted evolution in prokaryotes, which are often outer membrane protein genes or are involved in the invasion of the host immune system^9^, implying different evolutionary determinants in concerted evolution on different time scales.

### LRCE-DB: an online database to study concerted evolution

**Fig. 6:**
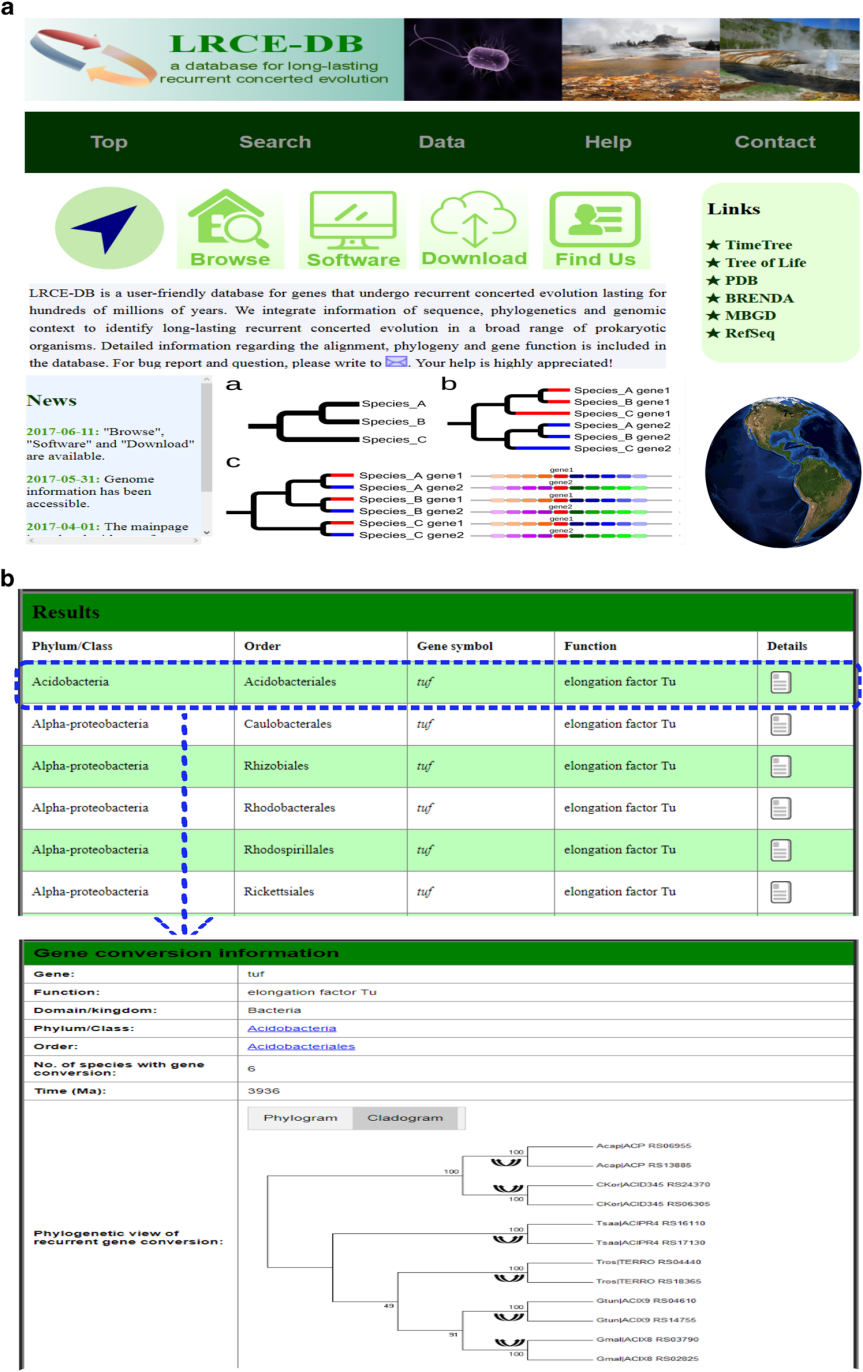
Examples of analysis using the LRCE-DB interface. **a** Homepage of the database. **b** Search results of concerted evolution in bacteria, and the view page of concertedly evolving genes (*tuf* in Acidobacteria)

Implemented with the goal of making the data easily accessible to interested researchers, we constructed an online web resource LRCE-DB (www.lrgcdb.eu) (Fig. 6a), which is the first online database designed for concerted evolution to the best of our knowledge. All data are deposited in MySQL database. The database web frontend was implemented in PHP5, HTML5, and CSS3, and was designed for Internet browsers on the basis of WebKit and derived layout engines. Users can browse genes by organism through the “Browse” interface. In the “Search” section, users can search genes of interest by gene name, taxonomy or the duration of concerted evolution (Fig. 6b). The graphical visualization of the phylogeny, sequence alignment, and other related information are available for each concertedly evolving gene (Fig. 6b). Moreover, users are provided the option to download the original data in batch by clicking on “Data” in the main toolbar (Fig. 6a).

## Discussion

In this study, we applied rigorous phylogenomic approaches to identify genes undergoing long-lasting recurrent concerted evolution in a broad range of prokaryotes. We excluded the possibility of independent duplication by integrating the information of gene synteny^15,16,31^. We also ruled out the possibility of convergent mutations in paralogs as a result of purifying selection at the amino acid level. In the case of strong purifying selection on the coding region of the genes, it would be expected that non-synonymous sites are similar whereas the synonymous sites are divergent between paralogs^7,32,33^. However, we observed high sequence similarity between paralogs at both synonymous and non-synonymous sites in most identified concertedly evolving genes (for alignments see www.lrgcdb.eu/Tree.php). This indicates that recurrent gene conversion is the main driving force that shapes the concerted evolution of the 26 genes identified in this study and it is likely ongoing^7^. Note that the two copies of *fla* were tandemly located, suggesting independent tandem duplication as an alternative possibility. The two copies of *mtrA* were also a pair of tandem duplicates. However, since the duplicate of *mtrA* has undergone a series of complex evolutionary scenarios including gene fusion and domain shuffling in all analyzed species, the high sequence similarity between *mtrA-1* and *mtrA-2* is unlikely to be due to independent tandem duplication in each lineage, as suggested by Wang et al. (2015)^12^.

Most previously reported concertedly evolving genes are found among species with relatively shallow phylogenetic depth^9^, which might overlook the long-term impact of concerted evolution on gene evolution. Our large-scale phylogenomic analysis suggests that long-lasting concerted evolution is exceedingly rare, but has played important roles in a small number of gene duplicates. While most duplicated genes may escape from concerted evolution over time, a few genes were found to be subjected to repeated sequence homogenization lasting for more than ~500 Ma. The findings of this study indicate the extremely long-term impacts of concerted evolution on the evolution of duplicated genes, and extend our understanding of the duration of concerted evolution to the scale of hundreds of millions of years, much longer than previously appreciated^7,10^. Note that the duration of concerted evolution can be overestimated if concertedly evolving genes are horizontally transferred rather than vertically inherited following an ancient duplication before the split of all analyzed species. This could be the case for *eftA* and *eftB*, as genes from species lacking concerted evolution were nested within those with concerted evolution, although lineage-specific gene loss as an alternative hypothesis cannot be rejected.

The recurrent pattern of gene evolution hints that it might not be a random process, but rather that it is favored by selection^13,34^. We speculate that concerted evolution may play a significant role in maintaining gene balance in a coadapted macromolecular complex and/or metabolic pathway. Sequence homogenization of paralogs as a result of concerted evolution can increase the concentration of a certain product when all gene copies are simultaneously expressed^35–39^. However, for a multisubunit complex, the alteration of the amount of only one subunit by concerted evolution might shift the reaction toward the formation of inactive subcomplexes, resulting in stoichiometric imbalance of the complex and deleterious effects on the cell^40–43^. This conundrum can be solved if all genes coding for the same complex undergo concerted evolution, as it can alter the amount of all subunits concertedly, maintaining the proper concentration of all subunits of the complex (Supplementary Fig. 9a). Our results indicate that 17 out of the 26 identified concertedly evolving genes encode genes from the same complexes and/or pathway (Tables 1 and 2). In addition, among the remaining nine genes, six encode proteins that can form homopolymers (*dxs*, *fla*, *tkt*, *ftsH*, *glnB*, and *archaeal histone*), whose stoichiometric balance should not be affected by the homogenization of paralogs of their encoded proteins. This idea can be best illustrated by the evolution of genes participating in the ammonia oxidation pathway (Figs. 2, 3; Supplementary Figs. 2, 3). Previous studies have shown that when one copy of *amoA* or *haoA* was inactivated, the other copies were more highly expressed to compensate for the loss of the first copy^44,45^. Also, the growth rate and the abundance of the AMO mRNA decreased by 25% and 37%, respectively, when *amoA-1* was inactivated in *Nitrosomonas europaea*^44^. The three single *haoA* mutant strains of *Nitrosomonas sp*. Strain ENI-11exibited 68% to 75% reduction of the wild-type growth rate^46^. These findings suggest that any single copy of the concertedly evolving paralogs is functionally important for maintaining the right dosage of the product^46^, and that concerted evolution may confer selective advantages in response to fluctuating ammonium availability in natural habitats^44^. Concerted evolution, in particular when it occurs only in coding regions, does not necessarily indicate high similarity in expression profile between paralogs, as found in yeast ribosomal proteins genes^47^. However, note that concerted evolution could result in the rapid spread of optimized codon usage, which in turn leads to dosage effects^38^. Moreover, even an increased dosage in certain conditions where it is especially important could confer considerable selective advantages, and drive long-lasting concerted evolution^36,48,49^. This might particularly be the case for prokaryotes, which are naturally exposed to changing environments.

Another mechanism that could cause gene imbalance is paralog interference, the process by which paralogs with divergent sequences interfere with each other by cross-interaction or competitive binding^50–52^. It would be tempting to infer concerted evolution as a mechanism to escape from paralog interference^52^ (Supplementary Fig. 9b). This idea is speculative due to the small number of identified concertedly evolving genes. However, there are several suggestive points. It was proposed that sequence homogenization by gene conversion was favored by selection for genes encoding proteins in ribosomes and nucleosomes in budding yeast since in tightly interacting complexes any change in one paralog might lead to deleterious effects in protein-protein interaction caused by paralog interference^16,47^. In support of this idea, 22 out of the 26 identified concertedly evolving genes encode proteins that are members of stable complexes. Furthermore, 1391 out of 4459 and 1151 out of 5915 genes in *Escherichia coli* and budding yeast, respectively, encode products that are members of protein complexes (Supplementary Data 2). This, suggests the potential enrichment of genes coding for members of complexes in genes undergoing long-lasting concerted evolution^16,47,53^.

While dosage imbalance and paralog interference affect the fates of duplicated genes in different ways, both of them can result in gene imbalance^50^. Because changes in gene balance follow directly after sequence homogenization of paralogs, concerted evolution by gene conversion or unequal crossover can confer instantaneous benefits by allowing beneficial mutations to rapidly spread, which does not require convergent mutations in all copies^10,39^.

Thus, we suggest that concerted evolution, which is likely the result of gene conversion followed by adaptive fixation, might be a mechanism for gene duplicates to maintain gene balance. Further analysis is needed to test this hypothesis. Our study focuses on ongoing concerted evolution that occurs across the full length of the gene. In future, it would be interesting to investigate cases of concerted evolution that occurred in part of the sequence over evolutionary time, but that is no longer ongoing^54,55^. Also, due to the abundance of genetic recombination and duplicated genes in eukaryotic genomes, it could be hypothesized that concerted evolution might be more common in eukaryotes; thus it will be interesting to examine whether the patterns found in prokaryotes hold true in eukaryotes.

In summary, our large-scale phylogenomic analysis identified 26 genes undergoing recurrent concerted evolution in a broad selection of prokaryotes, most of which have lasted for more than ~500 Ma and are likely still ongoing. We conclude that although long-lasting concerted evolution is exceedingly infrequent, it has clearly occurred and might have played significant roles in maintaining gene balance in many important pathways.

## Methods

### Selection of species

We carefully selected representative species used in the analysis based on the genomic data available at RefSeq. For species with multiple strains, only one strain was kept. For genera with more than five species, up to two species were chosen randomly as the representative species. Orders with fewer than six representative species were removed from subsequent analysis. Collectively, 682 species from 69 orders were analyzed in our study, and the information of their taxonomy and genomic sequences is available at www.lrgcdb.eu/Genome_info.php.

### Identification of long-lasting recurrent concerted evolution

We developed a bioinformatic pipeline iSeeCE (https://github.com/evolbeginner/iSeeCE) to perform large-scale identification of concertedly evolving genes based on rigorous phylogenomic methods (Fig. 1). We identified concerted evolution events in the unit of order. First, for species of each order, we retrieved protein sequences from NCBI RefSeq database (last accessed in April 2017) and clustered genes into families using OrthoMCL v2.0.4^56^. Because the result of OrthoMCL may be largely affected by the Markov Clustering (mcl) inflation index^56^, to minimize the bias in the classification of gene family, mcl was run using different inflation indices (1, 1.5, 2, 4, and 6) in OrthoMCL and the results were merged. Second, for each gene family, CDS sequences were aligned using MAFFT v7.043b^57^, and the phylogenetic tree was constructed with FastTree v2.1.7^58^, which uses heuristic algorithms to circumvent the low time efficiency in phylogeny reconstruction of large data sets, for an initial selection. In the initial selection, we selected all gene families where paralogs from the same species formed a monophyly in at least five species based on the phylogeny built by FastTree (Fig. 1). Third, for species in the same order, we identified syntenic orthologs supported by conserved gene synteny across species using Mauve^59^, as used in many studies^60–63^, assisted by custom scripts based on the best reciprocal BLAST hits^64^ and manual curation. Typically, at least three surrounding genes with orthologs across species were needed to support the synteny. Lastly, for each gene family that passed the initial selection, we manually checked members in the family, and constructed the phylogeny using RAxML v8.2.4^65^ with 500 bootstrap pseudoreplicates and GTR + GAMMA as the substitution model (-s input -n output -m GAMMAGTR -# 500 -p 123 -x 123 -f a).

We considered two paralogs from the same species as concertedly evolving genes if they i) formed a monophyly with bootstrap value of at least 70^66^, a widely accepted indication of support for a “real” clade^67,68^ ii) both have syntenic orthologs across species. Recurrent concertedly evolving genes were defined only if paralogs were found to undergo concerted evolution in at least five species. The species divergence time was estimated by TimeTree^69^. Phylogenetic trees were visualized using TreeGraph v2.5.0^70^.

### Information of protein complexes

The information of protein complexes of converted genes was manually collected by searching databases and literature, and is available in LRCE-DB (www.lrgcdb.eu). Protein complexes of *E. coli* and *S. cerevisiae* were retrieved from EcoCyc (https://ecocyc.org) and Yeast Complex Web (http://yeast-complexes.russelllab.org/complexview.pl?rm = download), respectively.

### Computer code

The computational pipeline iSeeCE is available at https://github.com/evolbeginner/iSeeCE. Other custom scripts are available at figshare under the DOI: 10.6084/m9.figshare.5732463^71^.

### Data availability

The data sets generated and analyzed during the current study are available in the online database LRCE-DB (www.lrgcdb.eu), as well as figshare under the DOI: 10.6084/m9.figshare.5732463^71^.

## Electronic supplementary material

Supplementary Information

Description of Additional Supplementary Files

Supplementary Data 1

Supplementary Data 2

## References

[1] Ohno S (1970). *Evolution by gene duplication*.

[2] Zhang JZ (2003). Evolution by gene duplication: an update. Trends Ecol. Evol..

[3] Lynch M, Conery JS (2000). The evolutionary fate and consequences of duplicate genes. Science.

[4] Taylor JS, Raes J (2004). Duplication and divergence: the evolution of new genes and old ideas. Annu. Rev. Genet..

[5] Xu GX, Guo CC, Shan HY, Kong HZ (2012). Divergence of duplicate genes in exon-intron structure. Proc. Natl Acad. Sci. USA.

[6] Liao DQ (1999). Concerted evolution: molecular mechanism and biological implications. Am. J. Hum. Genet..

[7] Nei M, Rooney AP (2005). Concerted and birth-and-death evolution of multigene families. Annu. Rev. Genet..

[8] Lawson, M. J., Jiao, J., Fan, W. G. & Zhang, L. Q. A pattern analysis of gene conversion literature. *Comp. Funct. Genomics* 761512 (2009). 10.1155/2009/761512 PMC2817553 20148076

[9] Santoyo G, Romero D (2005). Gene conversion and concerted evolution in bacterial genomes. FEMS Microbiol. Rev..

[10] Sugino RP, Innan H (2005). Estimating the time to the whole-genome duplication and the duration of concerted evolution via gene conversion in yeast. Genetics.

[11] Casola C, Conant GC, Hahn MW (2012). Very low rate of gene conversion in the yeast genome. Mol. Biol. Evol..

[12] Wang SS, Chen YH, Cao QH, Lou HQ (2015). Long-lasting gene conversion shapes the convergent evolution of the critical methanogenesis genes. Genes Genomes Genet..

[13] Kondrashov FA, Gurbich TA, Vlasov PK (2007). Selection for functional uniformity of tuf duplicates in gamma-proteobacteria. Trends Genet..

[14] Lathe WC, Bork P (2001). Evolution of tuf genes: ancient duplication, differential loss and gene conversion. FEBS Lett..

[15] Mansai SP, Innan H (2010). The power of the methods for detecting interlocus gene conversion. Genetics.

[16] Scienski K, Fay JC, Conant GC (2015). Patterns of gene conversion in duplicated yeast histones suggest strong selection on a coadapted macromolecular complex. Genome Biol. Evol..

[17] Garb JE, DiMauro T, Lewis RV, Hayashi CY (2007). Expansion and intragenic homogenization of spider silk genes since the triassic: evidence from mygalomorphae (Tarantulas and their kin) spidroins. Mol. Biol. Evol..

[18] Arp DJ, Chain PSG, Klotz MG (2007). The impact of genome analyses on our understanding of ammonia-oxidizing bacteria. Annu. Rev. Microbiol..

[19] Francis CA, Beman JM, Kuypers MMM (2007). New processes and players in the nitrogen cycle: the microbial ecology of anaerobic and archaeal ammonia oxidation. ISME. J..

[20] Sheikh AFEl, Poret-peterson AT, Klotz MG (2008). Characterization of two new genes, amoR and amoD, in the amo operon of the marine ammonia oxidizer *Nitrosococcus oceani* ATCC 19707. Appl. Environ. Microbiol..

[21] Norton JM, Low JM, Klotz MG (1996). The gene encoding ammonia monooxygenase subunit A exists in three nearly identical copies in Nitrosospira sp. NpAV.

[22] Nickelsen J, Rengstl B (2013). Photosystem II assembly: from cyanobacteria to plants. Annu. Rev. Plant. Biol..

[23] Watmough NJ, Frerman FE (2010). The electron transfer flavoprotein: ubiquinone oxidoreductases. Biochim. Biophys. Acta Bioenerg..

[24] Hahn FM (2001). 1-Deoxy-d-Xylulose 5-phosphate synthase, the gene product of open reading frame (ORF) 2816 and ORF 2895 in *Rhodobacter capsulatus*. J. Bacteriol..

[25] Kochetov GA, Solovjeva ON (2014). Structure and functioning mechanism of transketolase. Biochim. Biophys. Acta Proteins Proteom..

[26] Langklotz S, Baumann U, Narberhaus F (2012). Structure and function of the bacterial AAA protease FtsH. Biochim. Biophys. Acta Mol. Cell Res..

[27] Chevance FFV, Hughes KT (2008). Coordinating assembly of a bacterial macromolecular machine. Nat. Rev. Microbiol..

[28] Vanwonterghem I (2016). Methylotrophic methanogenesis discovered in the archaeal phylum Verstraetearchaeota. Nat. Microbiol..

[29] Hao B (2002). A new UAG-encoded residue in the structure of a methanogen methyltransferase. Science.

[30] Ferguson DJ, Gorlatova N, Grahame DA, Krzycki JA (2000). Reconstitution of dimethylamine:coenzyme m methyl transfer with a discrete corrinoid protein and two methyltransferases purified from Methanosarcina barkeri. J. Biol. Chem..

[31] Fawcett JA, Innan H (2011). Neutral and non-neutral evolution of duplicated genes with gene conversion. *Genes*.

[32] Eirín-López JM, González-Tizón AM, Martínez A, Méndez J (2004). Birth-and-death evolution with strong purifying selection in the histone H1 multigene family and the origin of orphon H1 genes. Mol. Biol. Evol..

[33] Rooney AP, Piontkivska H, Nei M (2002). Molecular evolution of the nontandemly repeated genes of the histone 3 multigene family. Mol. Biol. Evol..

[34] Stern DL (2013). The genetic causes of convergent evolution. Nat. Rev. Genet..

[35] Devis D, Firth SM, Liang Z, Byrne ME (2015). Dosage sensitivity of RPL9 and concerted evolution of ribosomal protein genes in plants. Front. Plant Sci..

[36] Hanikenne M (2013). Hard selective sweep and ectopic gene conversion in a gene cluster affording environmental adaptation. PLoS Genet..

[37] Moran Y (2008). Concerted evolution of sea anemone neurotoxin genes is revealed through analysis of the Nematostella vectensis genome. Mol. Biol. Evol..

[38] Sugino RP, Innan H (2006). Selection for more of the same product as a force to enhance concerted evolution of duplicated genes. Trends Genet..

[39] Innan H, Kondrashov F (2010). The evolution of gene duplications: classifying and distinguishing between models. Nat. Rev. Genet..

[40] Edger PP, Pires JC (2009). Gene and genome duplications: the impact of dosage-sensitivity on the fate of nuclear genes. Chromosome Res..

[41] Veitia RA (2004). Gene dosage balance in cellular pathways: implications for dominance and gene duplicability. Genetics.

[42] Conant GC, Birchler JA, Pires JC (2014). Dosage, duplication, and diploidization: clarifying the interplay of multiple models for duplicate gene evolution over time. Curr. Opin. Plant. Biol..

[43] Birchler JA, Veitia RA (2012). Gene balance hypothesis: connecting issues of dosage sensitivity across biological disciplines. Proc. Natl Acad. Sci..

[44] Hommes NG, Sayavedra-soto LA, Arp DJ (1998). Mutagenesis and expression of amo which codes for ammonia monoxygenase in Nitrosomonas europaea. J. Bacteriol..

[45] Hommes NG, Sayavedra-soto LA, Arp DJ (1996). Mutagenesis of hydroxylamine oxidoreductase in *Nitrosomonas europaea* by transformation and recombination. J. Bacteriol..

[46] Irota RH (2006). Transcriptional analysis of the multicopy hao gene coding for hydroxylamine oxidoreductase in Nitrosomonas sp. Strain ENI-11. Biosci. Biotechnol. Biochem..

[47] Evangelisti AM, Conant GC (2010). Nonrandom survival of gene conversions among yeast ribosomal proteins duplicated through genome doubling. Genome Biol. Evol..

[48] Kondrashov FA (2012). Gene duplication as a mechanism of genomic adaptation to a changing environment. Proc. R. Soc. Lond. B Biol. Sci..

[49] Kacar B, Garmendia E, Tuncbag N, Andersson DI, Hughes D (2017). Functional constraints on replacing an essential gene with its ancient and modern homologs. mBio.

[50] Panchy N, Lehti-Shiu M, Shiu SH (2016). Evolution of gene duplication in plants. Plant Physiol..

[51] Baker CR, Hanson-Smith V, Johnson AD (2013). Following gene duplication, paralog interference constrains transcriptional circuit evolution. Science.

[52] Kaltenegger E, Ober D (2015). Paralogue interference affects the dynamics after gene duplication. Trends Plant Sci..

[53] Ji X, Griffing A, Thorne JL (2016). A phylogenetic approach finds abundant interlocus gene conversion in yeast. Mol. Biol. Evol..

[54] Archibald JM, Roger AJ (2002). Gene conversion and the evolution of euryarchaeal chaperonins: a maximum likelihood-based method for detecting conflicting phylogenetic signals. J. Mol. Evol..

[55] Ishikawa SA, Kamikawa R, Inagaki Y (2015). Multiple conversion between the genes encoding bacterial class-I release factors. Sci. Rep..

[56] Li L, Stoeckert CJ, Roos DS (2003). OrthoMCL: identification of ortholog groups for eukaryotic genomes. Genome Res..

[57] Katoh K, Standley DM (2013). MAFFT multiple sequence alignment software version 7: improvements in performance and usability. Mol. Biol. Evol..

[58] Price MN, Dehal PS, Arkin AP (2010). FastTree 2-approximately maximum-likelihood trees for large alignments. PLoS ONE.

[59] Darling AE, Mau B, Perna NT (2010). ProgressiveMauve: multiple genome alignment with gene gain, loss and rearrangement. PLoS ONE.

[60] Nesbø C (2015). Evidence for extensive gene flow and Thermotoga subpopulations in subsurface and marine environments. ISME J..

[61] Bongrand C (2016). A genomic comparison of 13 symbiotic Vibrio fischeri isolates from the perspective of their host source and colonization behavior. ISME J..

[62] Kim JI (2017). Evolutionary dynamics of cryptophyte plastid genomes. Genome Biol. Evol..

[63] Qu XJ, Wu CS, Chaw SM, Yi TS (2017). Insights into the existence of isomeric plastomes in cupressoideae (Cupressaceae). Genome Biol. Evol..

[64] Goto N (2010). BioRuby: bioinformatics software for the Ruby programming language. Bioinformatics.

[65] Stamatakis A (2006). RAxML-VI-HPC: maximum likelihood-based phylogenetic analyses with thousands of taxa and mixed models. Bioinformatics.

[66] Hillis DM, Bull JJ (1993). An empirical test of bootstrapping as a method for assessing confidence in phylogenetic analysis. Syst. Biol..

[67] Soltis PS, Soltis DE (2003). Applying the bootstrap in phylogeny reconstruction. Stat. Sci..

[68] Holder M, Lewis PO (2003). Phylogeny estimation: traditional and Bayesian approaches. Nat. Rev. Genet..

[69] Hedges SB, Dudley J, Kumar S (2006). TimeTree: a public knowledge-base of divergence times among organisms. Bioinformatics.

[70] Stover BC, Muller KF (2010). TreeGraph 2: combining and visualizing evidence from different phylogenetic analyses. BMC. Bioinformatics..

[71] Wang, S. Genes that have undergone recurrent concerted evolution in Prokaryotes. 10.6084/m9.figshare.5732463.v2 (2017).

