# Supplementary material for: Phylogenomic analysis demonstrates a pattern of rare and long-lasting concerted evolution in prokaryotes
Source: Commun Biol. 2018 Feb 8;1:12. doi: 10.1038/s42003-018-0014-x (PMC6053082; doi:10.1038/s42003-018-0014-x)
Supplement: Supplementary file 1 — Supplementary Information [file 42003_2018_14_MOESM1_ESM.pdf]

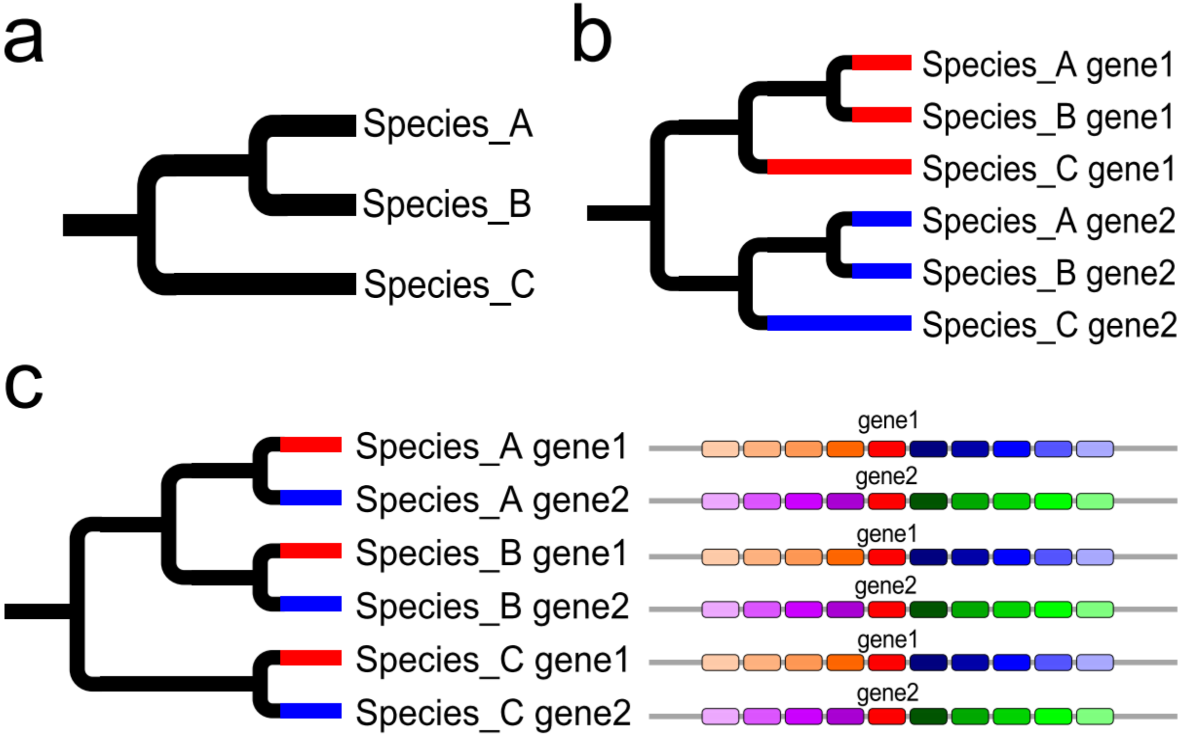

Supplementary Fig. 1. Schematic figure showing the identification of concerted evolution based on the combination of phylogeny and gene synteny. (a) The species tree. (b) The expected gene phylogeny in the absence of concerted evolution. (c) The expected gene phylogeny if concerted evolution occurs throughout all species.

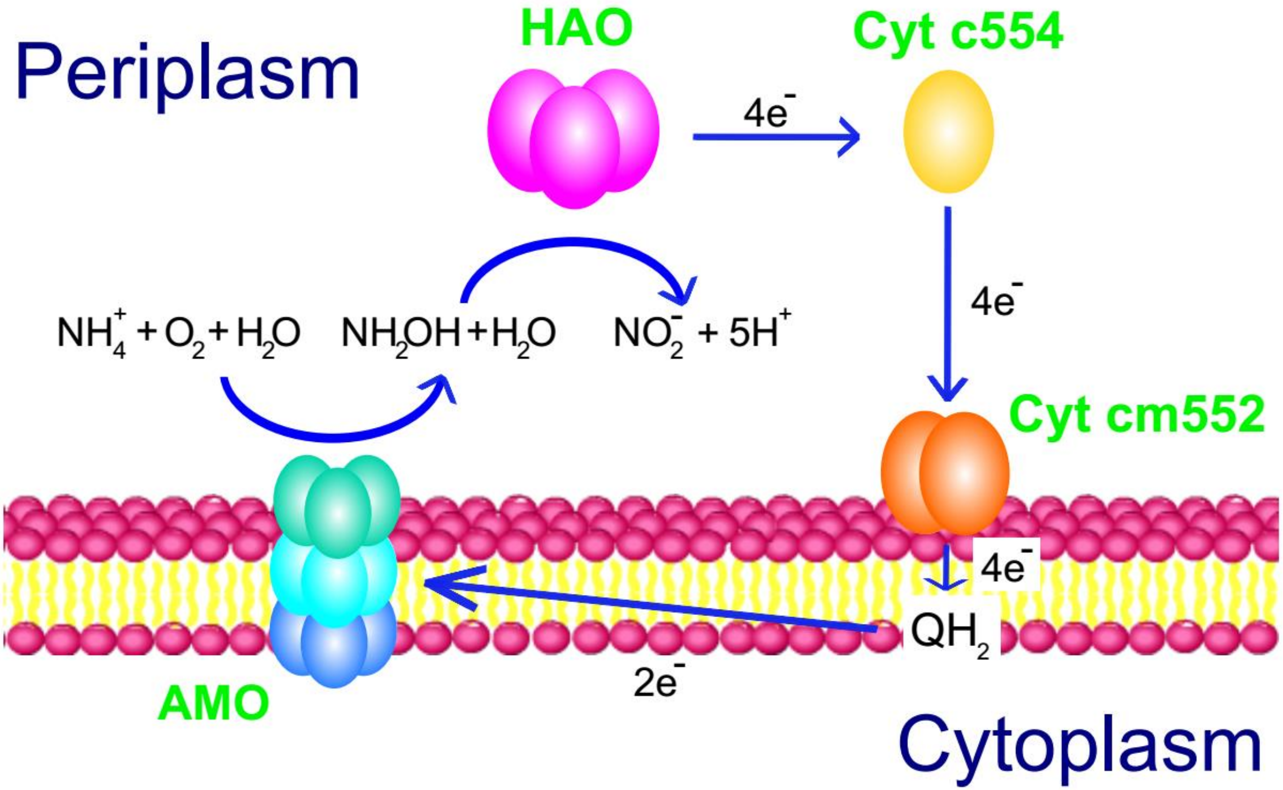

Supplementary Fig. 2. Graphical representation of the ammonia oxidation pathway in ammonia-oxidizing bacteria.

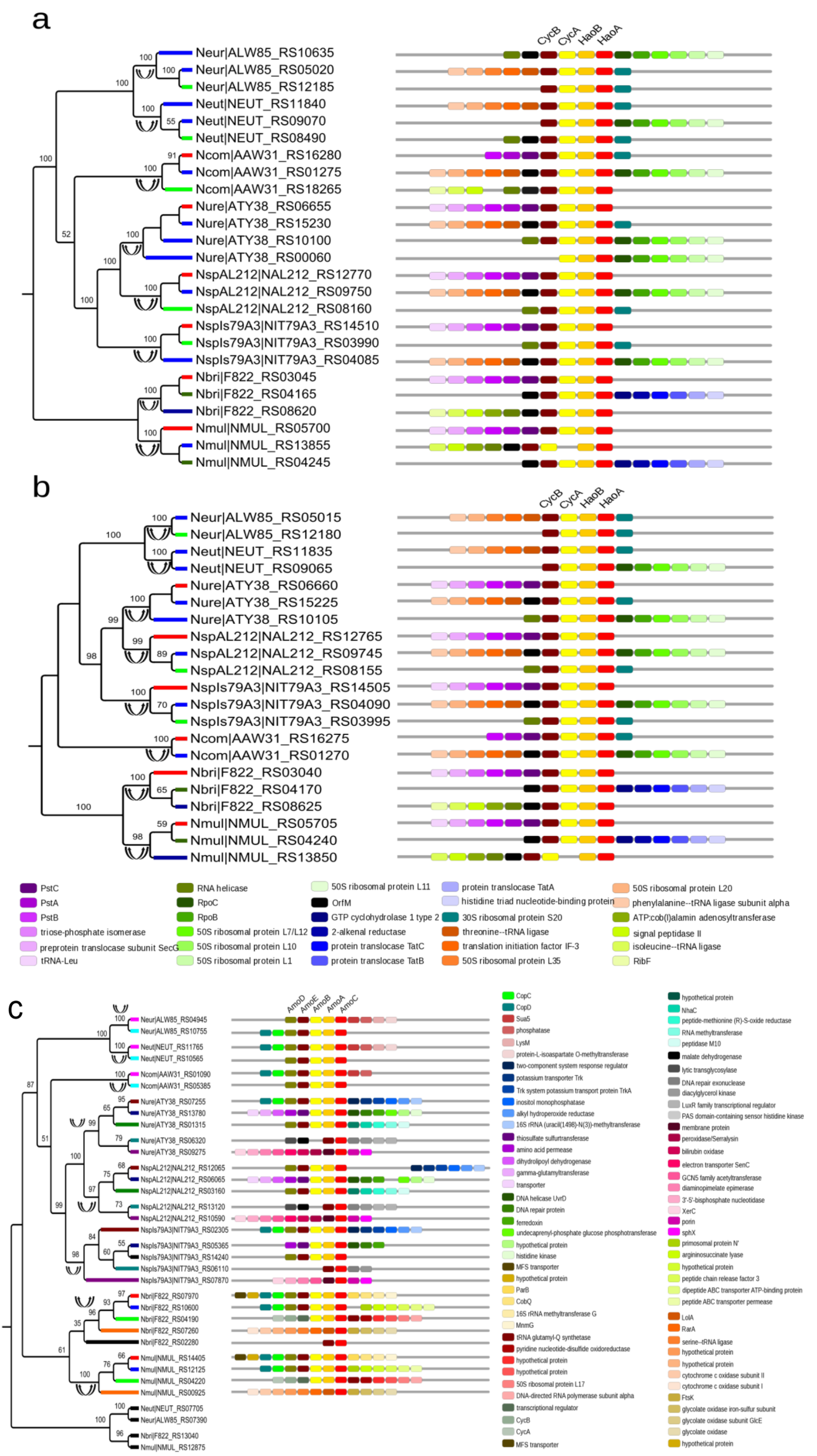

Supplementary Fig. 3. Phylogenetic trees of *cycA* (a), *cycB* (b), and *amoC* (c) in Nitrosomonadales. Double-headed arrows indicate concerted evoluion events. Syntenic orthologs are represented by thick branches in the same color in the phylogeny. Flanking genes are denoted by colored bricks, and chromosome segments are denoted by gray bars. Numbers adjacent to the nodes in the phylogeny are bootstrap percentages obtained from 500 pseudoreplicates. Only bootstrap percentages ⩾ 50 are shown. The name of each operational taxonomic unit is represented by the abbreviation of species name and gene locus. Abbreviations of species names are listed in Supplementary Data 1.

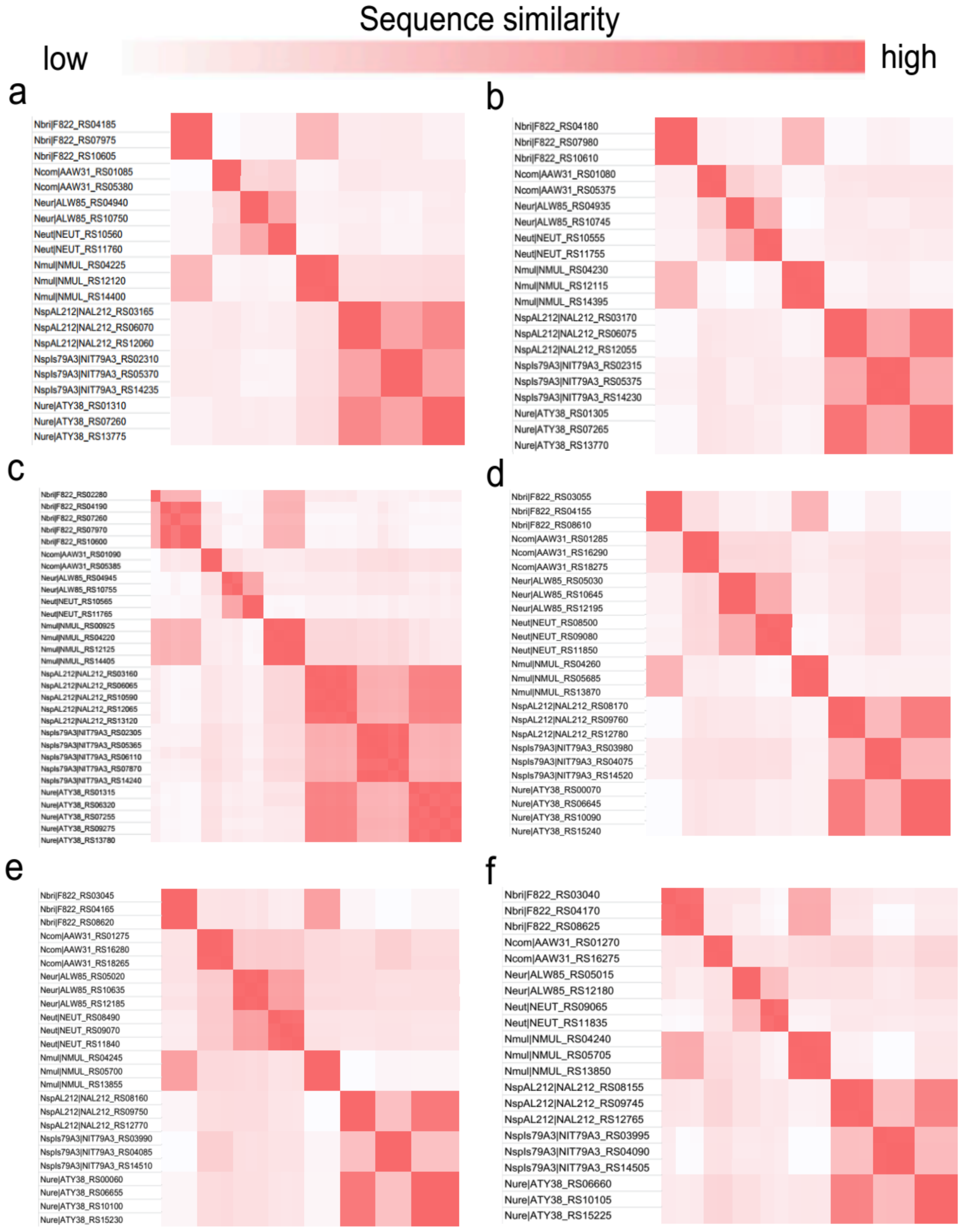

Supplementary Fig. 4. Heat maps of nucleotide sequence similarity for all pairwise combinations of homologs of genes known to be involved in ammonia oxidation pathway: *amoA* (a), *amoB* (b), *amoC* (c), *haoA* (d), *cycA* (e), and *cycB* (f). Colors are based on normalized nucleotide sequence similarity.

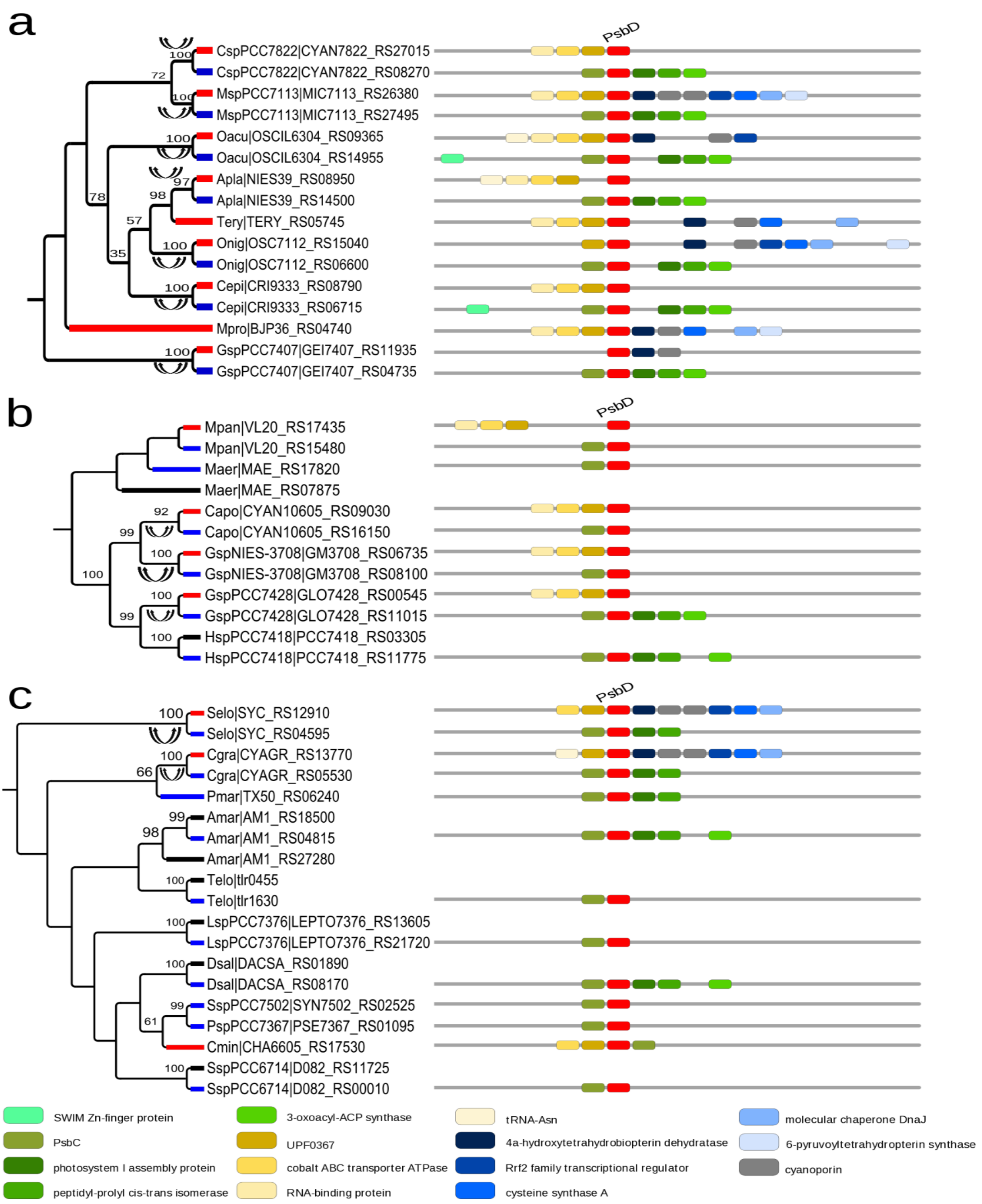

Supplementary Fig. 5. Phylogenetic trees of *psbD* in Oscillatoriales, Chroococcales and Synechococcales from cyanobacteria. Double-headed arrows indicate concerted evolution events. Syntenic orthologs are represented by thick branches in the same color in the phylogeny. Flanking genes are denoted by colored bricks, and chromosome segments are denoted by gray bars. Numbers adjacent to the nodes in the phylogeny are bootstrap percentages obtained from 500 pseudoreplicates. Only bootstrap percentages ⩾ 50 are shown. The name of each operational taxonomic unit is represented by the abbreviation of species name and gene locus. Abbreviations of species names are listed in Supplementary Data 1.

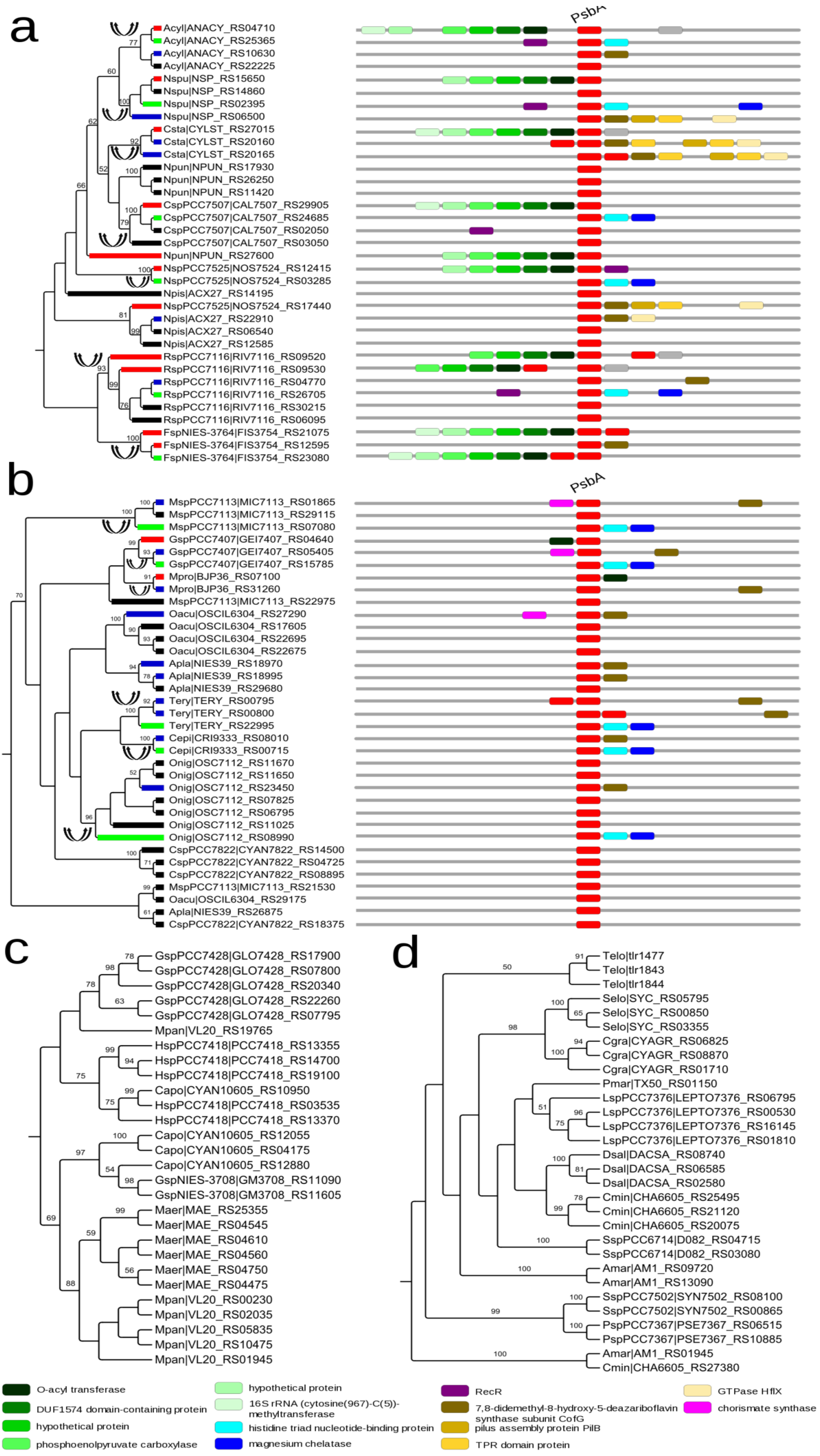

Supplementary Fig. 6. Phylogenetic tree of *psbA* in Oscillatoriales from cyanobacteria. Double-headed arrows indicate concerted evolution events. Syntenic orthologs are represented by thick branches in the same color in the phylogeny. Numbers adjacent to the nodes in the phylogeny are bootstrap percentages obtained from 500 pseudoreplicates. Only bootstrap percentages ⩾ 50 are shown. The name of each operational taxonomic unit is represented by the abbreviation of species name and gene locus. Abbreviations of species names are listed in Supplementary Data 1.

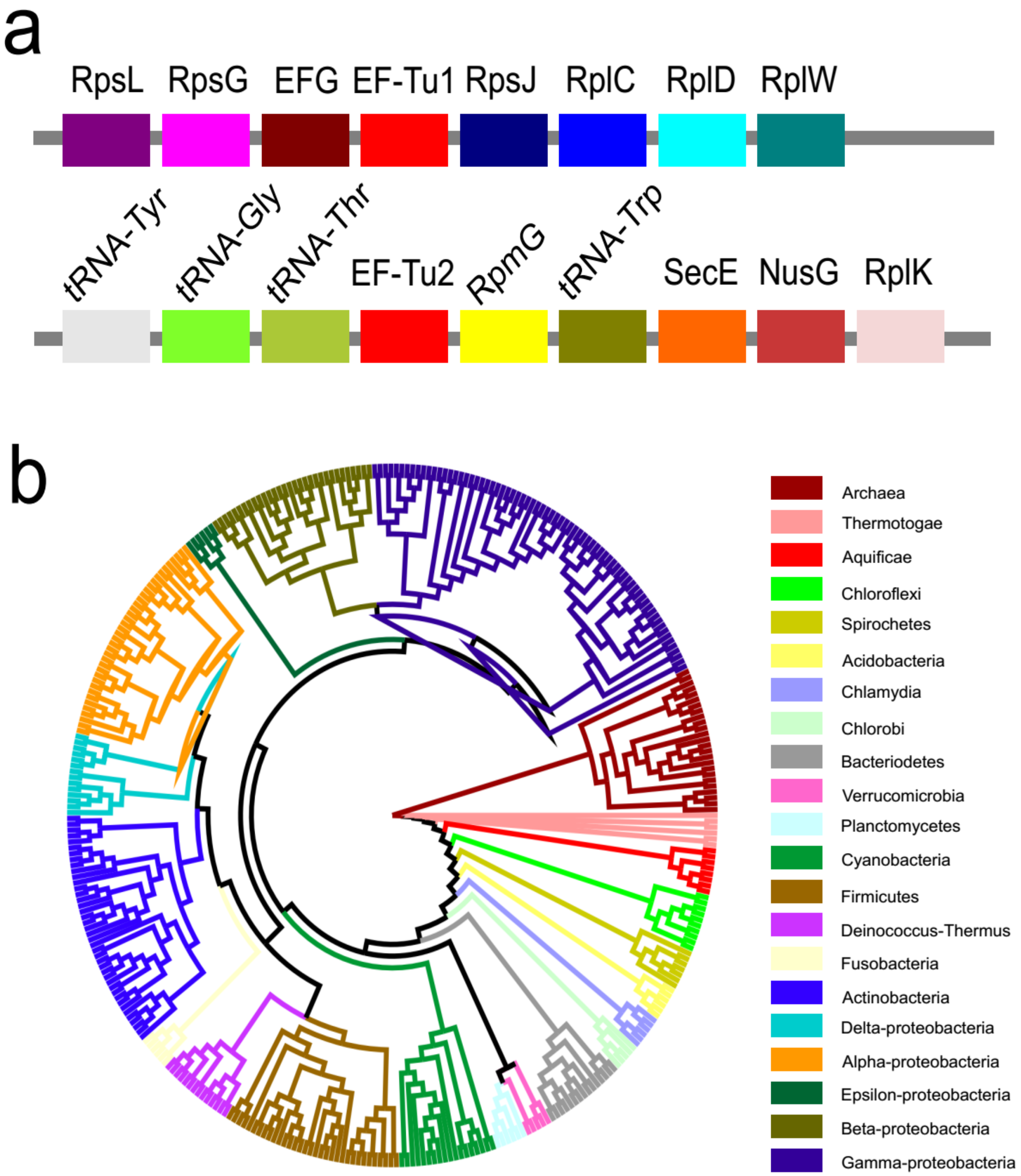

Supplementary Fig. 7. Genomic context and phylogeny of the gene encoding EF-Tu (*tuf*) across prokaryotes. (a) Genomic context of the gene encoding EF-TuA and EF-TuB. (b) The phylogeny of EF-Tu in prokaryotes constructed based on protein sequence alignment. The original tree and the alignment are available at figshare under the DOI: 10.6084/m9.figshare.5349145.

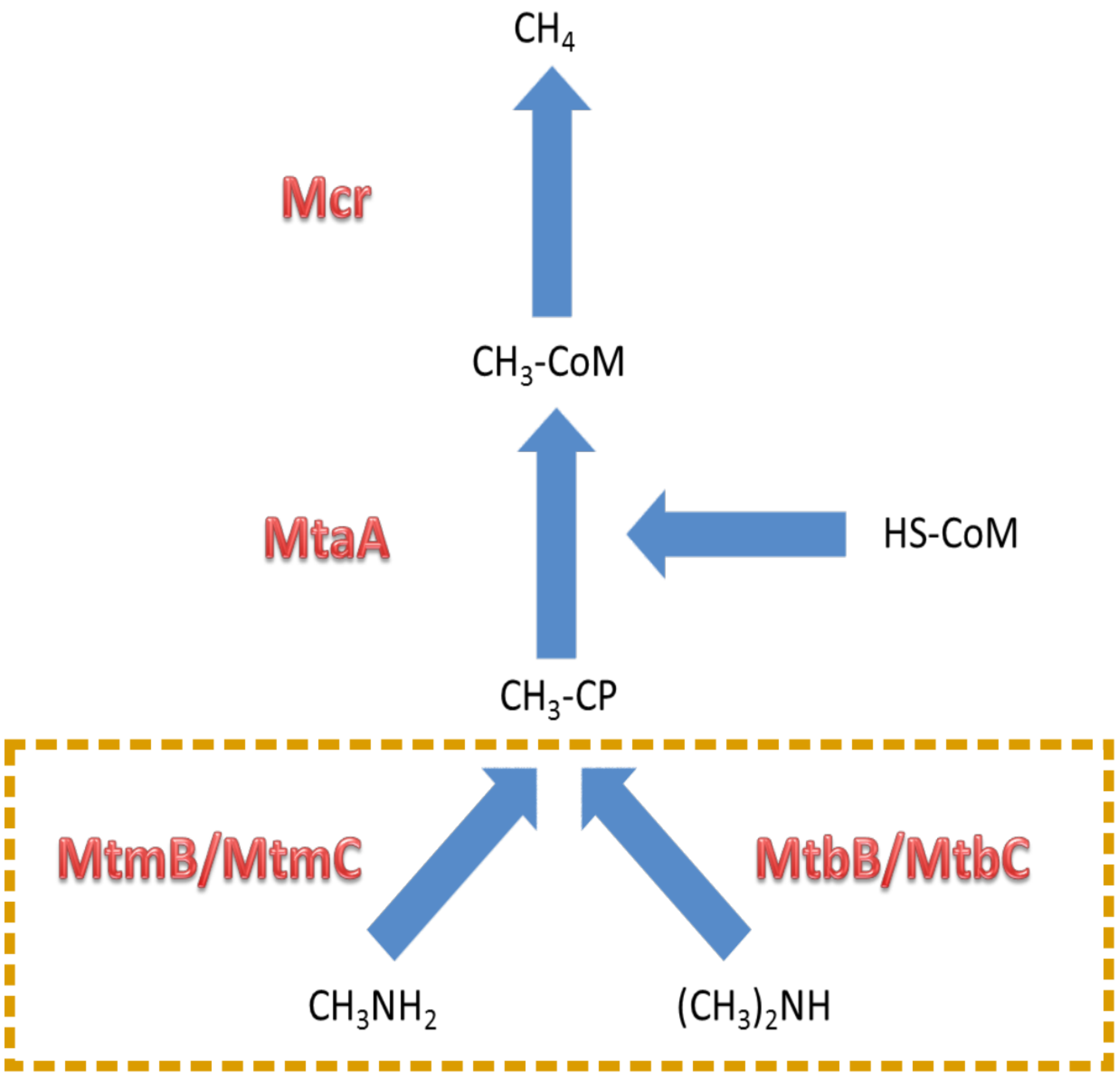

Supplementary Fig. 8. Schematic diagram of methylotrophic methanogenesis pathways from monomethylamine and dimethylamine.

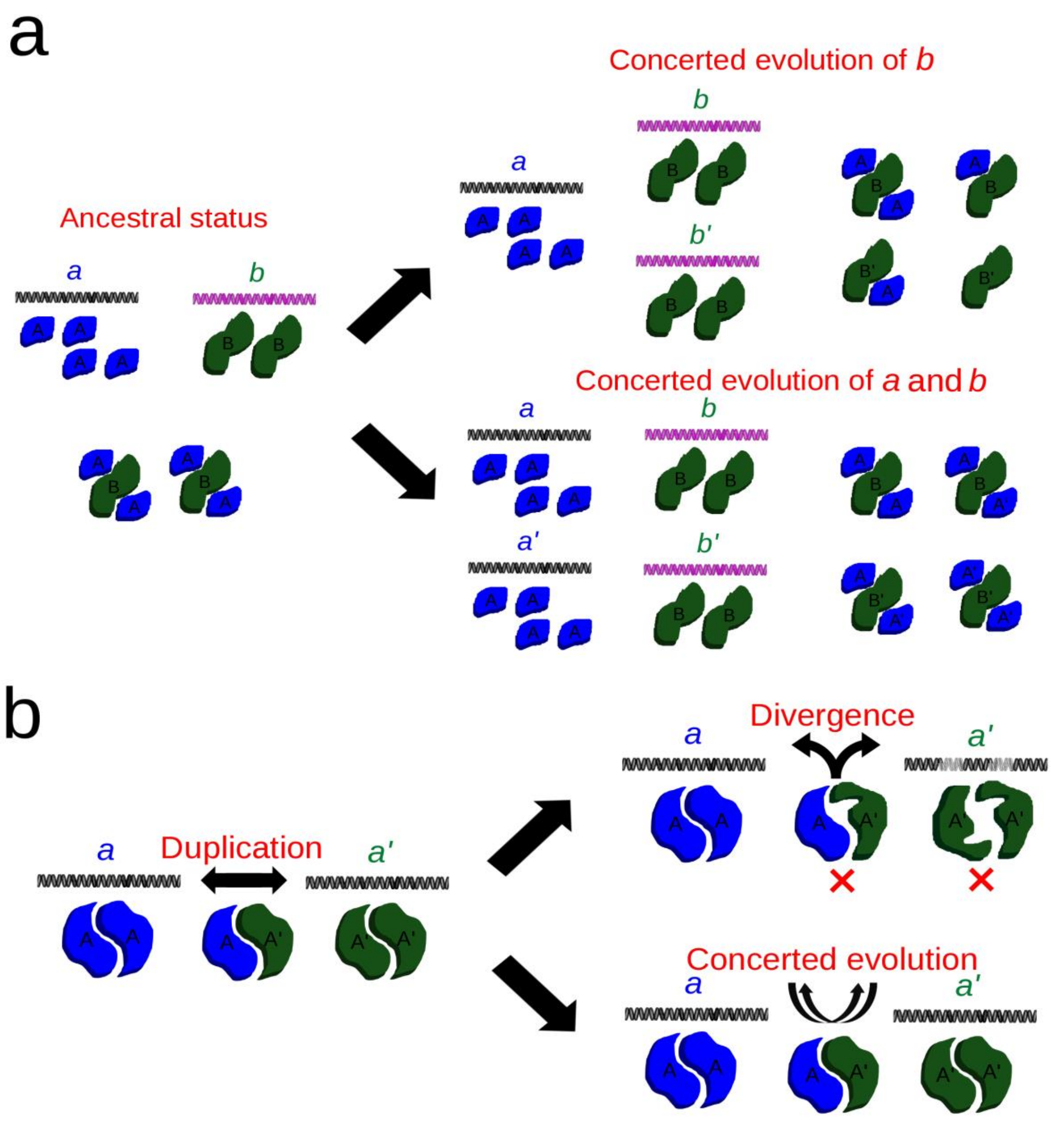

Supplementary Fig. 9. Models of the maintenance of gene balance by concerted evolution of paralogs of all genes in the complex. (a) The maintenance of dosage balance for genes encoding proteins in the same complex by concerted evolution. The products of gene *a* and *b* form a highly stable complex AB. By increasing the concentration of its product, concerted evolution following duplication of *b* produces inactive subcomplexes, resulting in the dosage imbalance. The concerted evolution of paralogs of both *a* and *b* ensures a proper concentration of the products, making the gene dosage rebalanced. (b) Escape of paralog interference by concerted evolution. The duplication of the ancestral gene *a* encoding a homodimer generates two duplicated genes. After duplication, the sequence divergence between the paralogs might cause paralog interference between *a* and *a'* by the formation of competitive inhibitors (AA') or inactive dimers (A'A'). Concerted evolution of *a* and *a'* could lead to the homogenization of the sequences, preventing the cross-interaction and releasing the duplicates from paralog interference.
