# Supplementary material for: Phylogenomic analysis demonstrates a pattern of rare and long-lasting concerted evolution in prokaryotes
Source: Commun Biol. 2018 Feb 8;1:12. doi: 10.1038/s42003-018-0014-x (PMC6053082; doi:10.1038/s42003-018-0014-x)
Supplement: Supplementary file 2 — Description of Additional Supplementary Files [file 42003_2018_14_MOESM2_ESM.docx]

**Description of Additional Supplementary Files**

File Name: Supplementary Data 1

Description: Abbreviations of species names.

File Name: Supplementary Data 2

Description: The lists of protein complexes of *Escherichia coli* and *Saccharomyces cerevisiae* (budding yeast).
